# Individualized approach to the surgical management of fibrous dysplasia of the proximal femur

**DOI:** 10.1186/s13023-018-0805-7

**Published:** 2018-05-02

**Authors:** Bas C. J. Majoor, Andreas Leithner, Michiel A. J. van de Sande, Natasha M. Appelman-Dijkstra, Neveen A. T. Hamdy, P. D. Sander Dijkstra

**Affiliations:** 1 Department of Orthopaedic Surgery, Centre for Bone Quality, Leiden University Medical Center, Postzone J11R, Postbus 9600, 2300 RD Leiden, The Netherlands; 2 Department of Medicine, Division of Endocrinology, Centre for Bone Quality, Leiden University Medical Center, Leiden, The Netherlands; 3 Department of Orthopaedic Surgery, Medical University of Graz, Graz, Austria

**Keywords:** Fibrous dysplasia, McCune-Albright syndrome, Proximal femur, Intramedullary nail, Blade plate

## Abstract

**Background:**

Fibrous dysplasia of the proximal femur presents with heterogeneous clinical manifestations dictating different surgical approaches. However, to date there are no clear recommendations to guide the choice of surgical approach and no general guidelines for the optimal orthopedic management of these lesions. The objective of this study was to evaluate treatment outcomes of angled blade plates and intramedullary nails, using as outcome indicators revision-free survival, pain, function and femoral neck-shaft-angle. Based on a review of published literature and our study findings, we propose a treatment algorithm, taking into account different factors, which may play a role in the selection of one surgical approach over another.

**Methods:**

Data were evaluated in thirty-two patients (18 male) from a combined cohort from the Netherlands and Austria, who had a surgical intervention using an angled blade plate (*n* = 27) or an intramedullary nail (*n* = 5) between 1985 and 2015, and who had a minimal follow-up of one year. The primary outcome was success of the procedure according to the revised Henderson classification. Secondary outcomes, which were assessed at one year and at the end of follow-up included: function (as measured by walking ability), pain and change in femoral neck-shaft angle over time.

**Results:**

Analysis of data showed that revision-free survival was 72% after a median follow-up of 4.1 years. Revision was necessary in two patients for structural failure due to a fracture distal to an angled blade plate and in 7 patients due to angled blade plate-induced iliotibial tract pain. At the end of follow-up 91% of all patients had good walking ability and 91% were pain free. There was no significant postoperative change in femoral neck shaft angle.

**Conclusion:**

Our data show that fibrous dysplasia of the proximal femur can be adequately and safely treated with angled blade plates or intramedullary nails, providing these are used according to specific characteristics of the individual patient. Based on published literature and our own experience, we propose an individualized, patient-tailored approach for the surgical management of fibrous dysplasia of the proximal femur.

**Electronic supplementary material:**

The online version of this article (10.1186/s13023-018-0805-7) contains supplementary material, which is available to authorized users.

## Background

Fibrous dysplasia is a genetic, non-inheritable, rare bone disorder that was first described in the late nineteen-thirties [1–3]. The disorder is due to a post-zygotic activating mutation of the GNAS-gene, which decreases GTPase activity of the stimulatory G-protein (G_s_α) [4, 5]. This results in increased intracellular levels of cAMP in bone forming cells, leading to local replacement of lamellar bone with ill-woven, under mineralized (fibrous) tissue of poor quality in affected parts of the skeleton, associated with clinical manifestations of pain, deformity and pathological fractures. The clinical spectrum of fibrous dysplasia varies widely, including single bony lesions (monostotic fibrous dysplasia), multiple lesions (polyostotic fibrous dysplasia), and the combination of polyostotic fibrous dysplasia with extra-skeletal manifestations such as café-au-lait skin patches and/or endocrinopathies such as precocious puberty and growth-hormone excess in the McCune-Albright Syndrome or intramuscular myxomas in the Mazabraud’s syndrome [6, 7]. The bony lesions are predominantly localized in the proximal femur and craniofacial bones [8]. Because of the weight-bearing forces acting on the lower extremities, the femur is most prone to deformities and fractures, ultimately resulting in the pathognomonic feature of fibrous dysplasia of the proximal femur; the ‘shepherd’s crook’ deformity [9].

The surgical management of fibrous dysplasia of the proximal femur has been particularly challenging due to the high load of mechanical forces acting at this skeletal site [10]. A number of surgical options have been originally proposed, including different types of bone grafting, various osteosyntheses, with or without additional osteotomies or a combination of these modalities. Over the past decade, however, there has been an increasing preference for the use of intramedullary nails and angled blade plates due to better treatment outcomes of these procedures [11–16]. In this study, we assess the clinical outcome of angled blade plates and intramedullary nails in fibrous dysplasia of the proximal femur, in a combined cohort of patients from the Leiden University Medical Center (LUMC) in the Netherlands and the Medical University of Graz (MUG) in Austria, using as outcome indicators implant function, revision-free survival and pain relief. We also perform a review of published literature on available surgical options in the management of fibrous dysplasia of the proximal femur, specifically focusing on the heterogeneity of the features of fibrous dysplasia at this site, and on the factors potentially affecting outcomes of the use of different procedures. Finally, based on our collective experience and on findings this study and on a review of published literature, we propose a patient-tailored approach for the surgical management of fibrous dysplasia of the proximal femur.

## Methods

### Patient selection

Ninety-six patients with an established diagnosis of fibrous dysplasia of the proximal femur who underwent surgery at the Orthopaedic Department of the LUMC or of the MUG between 1985–2015 were identified from the two hospitals’ registries. Included in the study were 32 patients who were treated with either an angled blade plate or an intramedullary nail and were followed-up for at least one year after surgery. The indications for treatment with an angled blade plate or an intramedullary nail were a symptomatic fibrous dysplasia lesion of the proximal femur extending beyond the femoral neck, a fracture with displacement and severe deformity of the proximal femur. Clinical and radiological data from the 32 patients included in the study were retrieved from their medical records.

Sixty-four patients in whom other surgical interventions were undertaken such as different types of grafting or other types of osteosyntheses were excluded from the study. Results from the group of patients from the LUMC who were treated with cortical allografts have previously been published [17]. Ethical approval was obtained from the Medical Ethics Committee of both centers participating in the study.

### Treatment protocol

According to the treatment protocol for fibrous dysplasia of the proximal femur followed at both the LUMC and the MUG, patients received an angled blade plate in case of a fracture with displacement, an (impending) fracture with involvement of the femoral shaft or in case of severe deformity of the proximal femur, in which case a valgus osteotomy was performed prior to implantation of the angled blade plate [17]. Only one patient from the LUMC received an intramedullary nail because the fibrous dysplasia lesion could not be bridged with an angled blade plate as the whole femur was affected (ID 17). Patients from the MUG were all initially treated with an angled blade plate as first choice, but the policy was changed to the use of intramedullary nails as first choice due to recurrent blade plate-induced pain of the iliotibial tract. The choice of additional bone grafting was based on the surgeon’s preference, particularly in the presence of relatively large lesions, although appreciating that the bone grafts would be likely to undergo resorption in time.

### Assessment of outcomes of surgical interventions

In this study the primary outcome of surgery using angled blade plates was success of the procedure, as defined by the modified Henderson classification for reconstructive surgery with endoprosthesis for bone tumours [18]. Secondary outcomes consisted of functional outcomes, improvement in pain and arrest of progression of femoral bowing and these outcomes were measured at three time points: directly after surgery (< 2 months), one year after surgery and at the end of the follow-up period. Data on functional outcome and pain were retrieved from the patients’ electronic medical records. Functional outcome was evaluated by assessing walking ability, which was categorized as good (walking a normal distance unaided and without complaints); moderate (able to walk only short distances) and severe (walking with the help of an aid (crutches/frame) or using a wheelchair). An increase in femoral deformity was evaluated by measured changes in the Femoral-Neck-Shaft-Angle (FNSA) of the femur on conventional radiographs.

### Statistical analysis

Statistical analysis was performed using SPSS for Windows, Version 23.0 (SPSS, Inc., Chicago, IL, USA). Results are presented as median and intermediate range or mean ± SD. FNSAs were analysed at different time-points using a general linear model for repeated measurements. Differences in FNSA-change between angled blade plates and intramedullary nails were analysed using an independent T-test.

## Results

### A. Combined cohort study

#### Patient characteristics

Individualized patients’ data and cohort characteristics are respectively shown in Tables 1 and 2. There was a slight predominance for the male gender (18 vs. 14) among the 32 patients from our combined cohort who were included in the study. Median age at diagnosis was 12 years (range 0–51 Whereas median age at surgery was 20 years (range 6–67 years), as many patients underwent surgery at age 20 or less (*n* = 16) as at the age of 21 years or older (*n* = 16), with 3 patients being younger than 12 years at time of surgery. Data on skeletal maturity were not available and as this may be delayed in the most severely affected young patients with FD, it is likely that some of the patients younger than 20 but older than 12 may have been skeletally immature at the time of surgery.

**Table 1:** Cohort Characteristics

	LUMC	MUG	Total
*N*	17	15	32
Male:Female	9:8	9:6	18:14
Median age at diagnosis (years (range))	9 (3–42)	23 (0–51)	12 (0–51)
Type of fibrous dysplasia			
Monostotic	3	12	15
Polyostotic	10	2	12
McCune-Albright	4	1	5
Type of surgery			
Angled Blade Plate	16	11	28
Intramedullary nail	1	4	5
Median age at surgery (years (range))	19 (11–67)	23 (6–51)	20 (6–67)
Preoperative fracture	77%	13%	47%
Characteristics of surgery			
Osteotomy	35%	13%	25%
Custom made	53%	0%	28%
Additional cancellous bone grafting	18%	53%	34%
Additional cortical strut grafting	53%	0%	28%
Median follow up after surgery (years)	4.1 (1–31)	4.7 (1–18)	4.1 (1–31)

**Table 2:** Individual patient characteristics

Patient ID	Gender/Age at Surgery	Type of fibrous dysplasia^a^	Prior surgery of the proximal femur	Indication for Surgery	Type of implant^b^	Osteotomy	Cancellous bone grafting	Cortical bone grafting	Follow-up in years	Failure
1	M/16	MAS	TEN Nails	Deformity	Custom made ABP	Yes	No	No	1,4	No
2	M/17	MAS	TEN Nails	Fracture	Custom made ABP	No	No	Yes	1,3	No
3	F/67	PFD	Fibular graft	Fracture	Custom made ABP	No	No	No	1,8	No
4	F/58	MAS	–	Fracture	Custom made ABP	No	No	Yes	2,0	No
5	M/12	PFD	Fibular graft, TEN Nails, external fixture	Fracture	Custom made ABP	No	No	No	4,1	No
6	M/29	PFD	Fibular graft (2×)	Pain	Custom made ABP	No	No	Yes	3,8	No
7	M/20	PFD	CBG, Plate osteosynthesis	Deformity	ABP	Yes	No	No	6,7	Yes
8	F/45	PFD	Fibular graft	Pain	Custom made ABP	No	Yes	No	4,1	No
9	F/19	MFD	Fibular graft	Fracture	ABP	No	No	No	4,7	No
10	F/18	PFD	Fibular graft (3×)	Deformity	ABP	Yes	Yes	Yes	9,4	No
11	M/16	MFD	–	Deformity	ABP	Yes	No	Yes	14,8	No
12	M/14	PFD	–	Deformity	ABP	Yes	No	Yes	17,3	Yes
13	M/11	PFD	–	Deformity	ABP	Yes	No	Yes	31,1	No
14	F/26	MFD	–	Fracture	ABP	No	Yes	Yes	1,8	No
15	F/40	MAS	Plate osteosynthesis, Fibular graft	Fracture	Custom made ABP	No	No	Yes	4,3	No
16	M/14	PFD	–	Fracture	ABP	No	No	No	1,0	No
17	F/30	PFD	Fibular graft	Pain	Custom made IMN	No	No	No	1,1	No
18	M/16	MFD	TEN Nails	Impending fracture	IMN	No	No	No	1,0	No
19	F/15	MFD	TEN Nails	Deformity	IMN	Yes	Yes	No	1,1	No
20	M/15	MAS	–	Deformity	IMN	Yes	No	No	1,4	No
21	M/51	MFD	–	Pain	ABP	No	No	No	7,4	No
22	F/21	MFD	–	Pain	ABP	No	Yes	No	7,8	Removal (irritation)
23	M/43	MFD	–	Impending fracture	ABP	No	Yes	No	3,6	Removal (irritation)
24	F/50	MFD	–	Impending fracture	ABP	No	No	No	4,0	Removal (irritation)
25	M/33	MFD	–	Pain	ABP	No	No	No	2,2	Removal (irritation)
26	F/29	MFD	–	Impending fracture	ABP	No	No	No	10,1	No
27	M23	PFD	–	Pain	ABP	No	Yes	No	15,9	No
28	M23	MFD	–	Pain	ABP	No	Yes	No	3,3	No
29	M14	MFD	–	Fracture	ABP	No	Yes	No	9,3	Removal (irritation)
30	F/46	MFD	–	Impending fracture	ABP	No	Yes	No	4,7	Removal (irritation)
31	F/6	MFD	–	Impending fracture	ABP	No	Yes	No	17,7	Removal (irritation)
32	M/13	PFD	Nancy Nails	Impending fracture	IMN	No	No	No	16,4	No

^a^*MAS* McCune-Albright Syndrome, *PFD* Polyostotic Fibrous Dysplasia, *MFD* Monostotic Fibrous Dysplasia

^b^*ABP* Angled blade plate, *IMN* Intramedullary nail

Fifteen patients had monostotic fibrous dysplasia, 12 had polyostotic fibrous dysplasia, 5 had McCune-Albright syndrome and one had Mazabraud’s syndrome. Fifteen patients (47%) had a fracture at a median of 3 years prior to surgery (range 0–43 years). Fourteen patients had surgery of the proximal femur prior to implantation of the angled blade plate or of the intramedullary nail, most commonly in the form of an allogeneic strut graft (*n* = 6) or of fixation using Titanium-Elastic-Nails (TEN) (*n* = 5). Primary indication for surgery included a disabling varus deformity (*n* = 8), fractures (*n* = 9), pain symptoms (*n* = 8) and impending fractures (*n* = 7). Twenty-seven patients received an angled blade plate compared to 5 patients who received an intramedullary nail. Eight patients (25%) needed an additional osteotomy. In 11 cases (34%) the fibrous dysplasia lesion was additionally filled with cancellous bone grafting or in 9 cases (28%) with allogeneic strut grafts. In 9 cases a custom-made titanium implant (8 blade plates and 1 intramedullary nail) was used. The procedure for implanting an angled blade plate was shorter in case an osteotomy was not required, compared to the time taken for implantation of an intramedullary nail (146 ± 46 vs. 230 ± 78 min). Procedures requiring an additional osteotomy took slightly longer to perform (respectively 182 ± 67 vs. 246 ± 85 min). Median follow-up after surgery was 4.1 years (range 1–31 years) for the whole cohort.

#### Revision-free survival

**Fig. 1:**
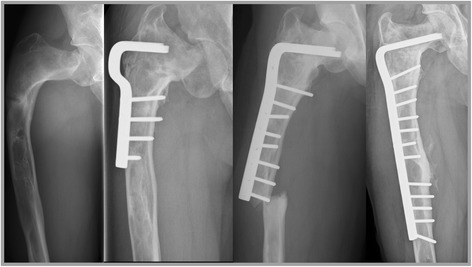
Structural failure after angled blade plate endoprosthesis. The first patient that needed revision surgery (ID 7) was a male with shepherd’s crook deformity of the femur and was previously treated elsewhere for a pathological femoral fracture by means of a valgus osteotomy combined with a short angled blade plate (Fig. 1). The coxa vara persisted however (FNSA 67°), associated with severe pain complaints for which he was referred to the LUMC, 8 years after his first surgery. A subtrochanteric osteotomy was performed and fixation was undertaken using a larger blade plate and a temporary external fixator, which resulted in improvement of the coxa vara (FNSA 97°) and good functional outcome. Pain was adequately controlled with additional bisphosphonate therapy. Four years later the patient unfortunately sustained a fracture of the femoral diaphysis, distal to the angled blade plate. This part of the femur was also affected with fibrous dysplasia and together with the stress riser of the distal angled blade plate formed a weak location in the femur, prone to fracturing. The angled blade plate was removed and a longer angled blade plate was inserted to cover the whole area of the affected femur. This procedure was followed by a good functional outcome and disappearance of pain symptoms lasting to the end of follow up

**Fig. 2:**
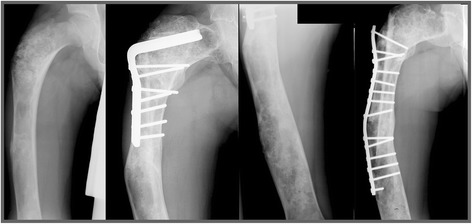
The second patient who needed revision surgery (ID 12) was a male with a fracture through a fibrous dysplasia lesion of the proximal femur who was treated with a correction osteotomy and fixation with an angled blade plate in combination with a allogeneic strut graft at the age of fourteen (Fig. 2). He unfortunately sustained a stress fracture distal to the blade plate, 3 years after the initial surgery. The angled blade plate was removed during revision surgery and a long femoral plate was used to stabilize the femoral shaft. The patient was able to walk with crutches and had no more pain complaints. However, his severe coxa vara (FNSA 69°) remained unchanged

Two patients needed revision surgery 3 and 4 years after initial surgery due to structural failure of the implant (Henderson type 3B), in both cases caused by a fracture distal to the implanted angled blade plate (Figs. 1 and 2). None of the other 30 patients included in this study sustained a fracture after surgery. Seven patients with monostotic fibrous dysplasia who were primarily treated with an angled blade plate in combination with cancellous bone grafting had soft tissue failure (Henderson type 1A) in the form of persistent iliotibial tract complaints requiring removal of the angled blade plate in all after a median of 3.0 (range 0–5) years after initial surgery. All 7 patients became pain free after removal of the endoprosthesis and none had recurrence of pain, fractures, or required further surgery for the duration of follow-up. None of the 32 patients had neurovascular complications, complications related to the osteosyntheses or post-operative infections. Revision-free survival was thus 97% for the whole cohort after 1 year and 72% at the end of follow-up, a median of 4.1 years (range 1–31 years) after surgery.

#### Pain symptoms and functional outcome

Thirty of the 32 patients (94%) had pain at the site of the fibrous dysplasia lesions of the proximal femur prior to surgery. Only six of these 30 patients had persistent pain at this site one year after surgery, so that 81% of all patients were pain free at this time point. This figure further increased to 91% at the median end of follow-up of 4.1 years post-operatively. Prior to surgery only 16% of the 32 patients had a good function (walking a normal distance unaided and without complaints), 66% had moderate function (able to walk for a small distance) and 18% could only mobilize with the help of crutches or frame or by using a wheelchair. One year postoperatively 88% had good function and at the end of follow-up 29 of the 32 patients (91%) of patients could walk a normal distance unaided. Three patients still needed crutches at the end of follow-up and one patient with mental retardation was wheelchair bound.

#### Femoral neck shaft angle

In the group of patients who required an additional valgus osteotomy, average FNSA was corrected from 89° ± 20 to 118° ± 13 directly after surgery. In the whole cohort, mean FNSA was 123.1° ± 11 after implantation of an angled blade plate and 131.3° ± 1 after implantation of an intramedullary nail. FNSA did not significantly further change one year after surgery or at the end of follow-up (*p* = 0.129). There was no significant difference between patients who received an angled blade plate compared to those receiving an intramedullary nail regarding change of FNSA after one year (*p* = 0.541) or at the end of follow-up (*p* = 0.591). There was also no difference in FNSA change after one year (*p* = 0.275) or at the end of follow-up (*p* = 0.207) between patients with or without additional bone grafting.

### B. Literature review of surgical procedures used in fibrous dysplasia of the proximal femur

#### Curettage and bone grafting

Historically, fibrous dysplasia lesions of the proximal femur were treated by curetting the lesion, with or without filling of the emptied cavity with cancellous bone grafts. This technique was soon found to be highly inefficient due to high recurrence rates and is therefore no longer used (Table 3) [10, 11, 13, 19–23]. In 1986 Enneking and Gearen [24] suggested the use of allogeneic strut grafts instead of cancellous bone grafts, arguing that cortical allogeneic bone was less likely to be resorbed than cancellous bone and would therefore offer more efficient and especially long lasting stability to fibrous dysplasia lesions of the proximal femur. However, other workers later reported that bone grafting often leads to resorption of the graft and consequently to failure of the procedure in patients with fibrous dysplasia, who subsequently have to be subjected to revision surgery (Table 3) [17, 23, 25, 26]. Different factors may play a role in graft survival between studies. Patients with no fracture preoperatively have been shown to have a good prognosis with allogeneic strut grafts, providing that there is sufficient healthy bone proximally in the femoral neck for the strut graft to be anchored and to grown into [17]. Putting these findings together, it may be concluded that there is a place for allogeneic strut grafts in the management of impending fractures and of pain due to fibrous dysplasia of the proximal femur in selected cases in which there is no history of a pathological fracture of the proximal femur, there is enough bone stock proximal in the femoral neck to anchor the strut graft, there is no indication for a valgus osteotomy and the fibrous dysplasia lesion does not extend to the femoral shaft. Cortical grafts should not be used in patients in whom one or more of these risk factors are identified in order to avoid graft resorption, failure and the need for revision surgery. Based on experience in the LUMC (unpublished data), revision surgery with a second allogeneic strut graft should not be recommended in a previously treated femur as this is prone to fail (Additional file 1).

**Table 3:** Overview of the literature on surgical treatment of fibrous dysplasia of the proximal femur

Type of Surgery	Author/Year	*N*	Type of Surgery	Mean Follow-up	Failure	Outcome
Grafts	Harris et al. (1962) [10]	10	Cancellous Autograft	Unknown	5/10	Poor in 50%
	Nakashima et al. (1984) [21]	8	Autograft (unknown origin)	Unknown	2/8	Poor in 25%
	Enneking er al. (1986) [24]	15	Cortical Autograft	6 years	2/15	Poor in 2out of 15 (revision surgery)
	Stephenson et al. (1987) [20]	18	Cancellous Autograft	10.4 years	25/31	Poor in 81%
	Guille et al. (1998) [19]	22	Cancellous Autograft	15 years	22/22	Resorption of graft in 100%
	Ippolito et al. (2003) [11]	5	Cancellous Autograft*	Unknown	3/5	Poor in 60% (revision surgery)
	George et al. (2008) [25]	8	Cortical Autografts	4.1 years	1/8	Poor in 12.5% (recurrence).
	Tong et al. (2013) [27]	13	Cancellous Autograft with internal fixation	12–32 months	0/13	No patients required revision surgery
	Kushare et al. (2014) [13]	8	Various Grafts	3 years	Unknown	Unclear
	Nishida et al. (2015) [26]	8	Cortical Autograft with compression hip screw	75 months	0/8	No patient had poor outcome
	Leet et al. (2016) [23]	46	Various Grafts	19.6 years	39/52	KM-survival: 50% survival at 14.5 years
	Majoor et al. (2016) [17]	28	Cortical Allograft	13 years	13/28	Good outcome in patients without a preoperative fracture and adequate proximal anchoring
Intramedullary Nail	Harris et al. (1962) [10]	3	Single intramedullary rod	Unknown	1/3	The only patient with fibrous dysplasia of the collum developed a severe varus deformity
	Freeman et al. (1987) [29]	6	Multiple osteotomies with a Zickel Nail	34.5 months	2/6	Two patients needed revision surgery.
	Keijser et al. (2001) [22]	5	Intramedullary nails, additional multiple osteotomies in one patient	19.4 years	3/5	Three patients needed at least one revision surgery after the first IMN.
	O’Sullivan et al. (2002) [39]	10	Bilateral osteotomies and Sheffield rods	18 months	3/10	Three femurs needed revision surgery. 4/5 patients had a bad functional outcome due to severe coxa vara.
	Ippolito et al. (2003) [11]	19	Interlocking cephalomedullary nails	Unknown	0/19	All patients had a good outcome with no worsening of deformities
	Jung et al. (2006) [30]	7	Multiple osteotomies with intramedullary nails	30 months	0/7	No patients needed a revision surgery and good functional outcome in all patients
	Yang et al. (2010) [14]	14	Valgus osteotomy with intramedullary nails	75.3 months	0/14	No patient needed revision surgery
	Zhang et al. (2012) [32]	28	IMN, additional osteotomy in 8 patients	50 months	0/28	No patients needed revision surgery. Good functional outcome in the majority
	Kushare et al. (2014) [13]	16	Intramedullary nails	3 years	1/16	One patient required further surgery and 5 had pain at last follow-up
	Ippolito et al. (2015) [16]	11	Two stage coxa vara correction and definitive fixation with an interlocking nail	4.7 years	4/11	Four patients had complications after the first surgery and another four needed further surgery after the second implant.
	Benedetti Valentini et al. (2015) [15]	8	Customized adult humeral nail in children (4–7 years)	2.9 years	3/8	Three patients required revision surgery as an adult. One patient required distal screw removal and acquired nail breakage
	Present study	5	Intramedullary nails, one of which was customized	4.1 years	0/5	All patients had a good outcome with no worsening of deformities
Angled Blade Plate	Ippolito et al. (2003) [11]	2	Angled blade plates after valgus osteotomy	4.5 years	1/2	One failed due to cutting out of the plate. The other ABP had a good outcome.
	Leet et al. (2016) [23]	2	Angled Blade Plates	Unknown	Unknown	Outcome of ABP not described
	Ippolito et al. (2015) [16]	8	Angled Blade Plates	Unknown	1/8	One patient had screw loosening with lateralization of the plate
	Present study	28	Angled Blade Plates, 8 of which were customized	4.1 years	2/28	Two failures, in 7 patients ABP removed due to complaints of the iliotibial tract
Dynamic/Compression Hip Screw	Li et al. (2013) [34]	21	Valgus osteotomy with DHS fixation	19–128 months	2/21	One patient revision surgery with an intramedullary nail after a fracture and one had a loose lag screw.
	Tong et al. (2013) [27]	2	Valgus osteotomy with DHS fixation	12–32 months	0/2	No patient needed revision surgery
	Nishida et al. (2015) [26]	8	Cortical Autograft with compression hip screw	75 months	0/8	No patient needed revision surgery

A number of studies have reported the use of different types of bone grafts combined with internal fixation [11, 23, 26, 27]. In our cohort there was no difference in outcomes between patients with or without simultaneous bone grafting. To our knowledge, the advantage of additional bone grafting has never been analyzed in detail so that it is difficult to interpret whether the good outcomes in some of these studies were due to the additional bone grafting or were solely due to the beneficial stabilizing effect of the mechanical implant.

#### Intramedullary nail

**Fig. 3:**
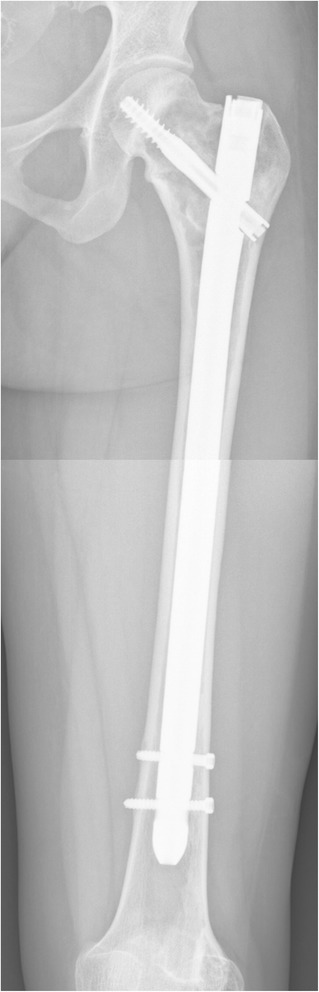
Customized intramedullary nail. A customized intramedullary nail with a HA-coated proximal screw was used in one patient (ID 18), in order to ensure ingrowth in the femoral neck. The nail had an enlarged diameter because this particular patient had severe cortical thinning throughout the length of the femur, providing insufficient structural support for a standard sized intramedullary nail

Over the past decade there has been a preference for using intramedullary nails in the management of fibrous dysplasia of the proximal femur [10–12, 14–16, 22, 28–33]. Despite apparent consensus in the literature about this surgical modality, there is still much debate on the type of intramedullary nail that should be used. Solitary rods, lacking proximal and distal locking and therefore failing to offer sufficient support, frequently lead to persistent coxa vara deformity and poor functional outcome, suggesting they should not be used in fibrous dysplasia of the proximal femur (Table 3). However, cephalomedullary nails with bipolar fixation of both the proximal and distal end of the implant have demonstrated good outcomes in terms of low failure rates and good function in patients with severe forms of fibrous dysplasia, as they provide sufficient support to the proximal femur [11, 13, 14, 16, 30, 32]. Compared to angled blade plates, intramedullary nails offer the advantage of being minimally invasive and being more frequently used in general trauma units, which generates more experience with their use, providing an osteotomy is not required. Although fractures have also been reported after treatment with intramedullary nails, they are generally believed to associated with a lower risk of developing fractures compared to angled blade plates [16, 22]. In the current study, we also demonstrate a good functional outcome in 5 patients who were treated with an intramedullary nail, with no revision surgery required, good walking ability and complete relief of pain after up to 16 years of follow-up (Fig. 3). Based on these findings, intramedullary nails appear to be a sound treatment option for fibrous dysplasia of the proximal femur, providing that a bipolar proximal and distal fixation of the nail can be performed and that the femoral neck screw does bridge the lesion in the metaphysis.

#### Angled blade plates

The use of angled blade plates in the management of fibrous dysplasia of the proximal femur has been reported in a number of studies, all conducted in small numbers of patients, almost always using different types of implants and lacking reporting on functional outcomes [11, 16, 23]. This scarcity of reported data with the use of this implant modality precludes the drawing of any firm conclusion on the use of the angled blade plates in fibrous dysplasia of the proximal femur. In the present study we demonstrate that angled blade plates have a good outcome in the majority of cases of fibrous dysplasia of the proximal femur with a low postoperative fracture rate and arrest of progressive varization of the femur. In our series only two out of the 27 cases (7%) treated with an angled blade plate developed a fracture. In both cases the fracture occurred distal to the angled blade plate, probably because of failure of the angled blade plate to completely cover the fibrous dysplasia lesion. Because the distal part of the plate may function as a stress riser, which may in itself increase fracture risk in the presence of a fibrous dysplasia lesion, we do recommend that to avoid this complication, the angled blade plate is positioned to bridge the entire fibrous dysplasia lesion. The implant positioning should also ensure that both proximal en distal ends are anchored into healthy bone. To avoid these complications we have been recently using customized angled blade plates in the LUMC. These customized blade plates can be designed to cover the whole of the affected part of the femur and may thus more efficiently prevent fractures. Based on published literature and on our two centers’ experience, it is our opinion that angled blades plates hold an advantage over intramedullary nails in patients with severe deformities of the femur shaft and thus of the intramedullary canal. Severe deformity often necessitates performing multiple difficult osteotomies, which precludes the introduction of an intramedullary nail, while an angled blade plate can still be easily positioned to ensure stability of the fibrous dysplasia lesion as customized angled blade plates in these cases accurately follow the curves of the deformed femur and this provides adequate fitting and stability to the femur. Angled blade plates do also hold an advantage over intramedullary nails in patients with previous metaphysical cortical grafts, as the partially resorbed cortical bone often does not allow placement of the proximal screws of an intramedullary nail while the angled blade plate can still be implanted with relative ease thus providing adequate mechanical support. A possible downside of the angled blade plate that has come to light in our study is the possibility of developing complaints of the iliotibial tract such as pain and associated difficulty in walking. Because the 7 angled blade plate recipients who developed these complaints in our study originated from the same center, the question arises whether the surgical technique used differed between our two centers. A closer look revealed that in the LUMC, where none of these patients developed complaints of the iliotibial tract, an additional step in the procedure was for the 95-degree angle in the cortex to be milled out to ensure submerging of the plate into the bone. In the MUG, removal of the hardware in patients with complaints of the iliotibial tract resulted in good functional outcome in all with no reported (new) pathological fractures. Notwithstanding these outcomes may have also be due to the fact that these cases had mild fibrous dysplasia and it is likely that the femur in more severe types of fibrous dysplasia may not be so forgiving after removal of any form of supporting hardware. Based on published literature and data from our combined cohort we can conclude that angled blade plates can be effectively and safely used in fibrous dysplasia of the proximal femur, providing that the lesion can be adequately bridged by the implant with proximal and distal locking of the angled blade plate into healthy bone. Based on the LUMC experience, we also recommend that the surgeon should ensure submerging of the plate into the bone, as this appears to prevent complaints of the iliotibial tract.

#### Dynamic/compression hip screw

A number of studies have reported the use of dynamic hip screws (DHS), either after a valgus osteotomy or for stabilization of a pathological fracture of the proximal femur [27, 34]. Nishida et al. used a compression hip screw (CHS) in combination with an allogeneic strut graft in 8 patients, which led to similar results after a mean follow-up of 75 months [26]. However, studies addressing the use of these devices in the treatment of pathological fractures show that they have a high failure rate compared to angled blade plates and intramedullary nails [35, 36]. Additionally, the short stem of the DHS and of the CHS does not seem to be able to protect the distal part of the femur, which is often affected in fibrous dysplasia, from fracturing. We would therefore not advocate the use of the DHS or CHS in fibrous dysplasia of the proximal femur.

#### Challenges of surgery of the proximal femur in pediatric fibrous dysplasia patients

Treatment of pediatric patients with fibrous dysplasia of the proximal femur calls for a different surgical approach to that of adults with this disease localization, as the growth of the femur has to be accounted for in the placement of internal fixation to avoid damage to the growth plate or to the pediatric vascular circulation of the proximal femur [9, 11, 37]. Moreover, standard intramedullary devices used in adults will generally not fit into the small femoral shaft of children, ruling out their use in most young children, especially in growing patients with an open physis [15]. Titanium elastic nails (TEN) have frequently been used to address fractures, and although most fractures show good healing, the TENs will not prevent any subsequent fracture or the progression of deformities and should therefore not be used in the proximal femur of young patients with fibrous dysplasia [11, 15]. Different intramedullary devices have been proposed, among which humeral nails and a new small diameter pediatric interlocking intramedullary device [15, 23]. However, there are to date scarce data on the use of these devices and it has been associated with a number of drawbacks such as continuous deformation of the femur and introduction of the nails into the apophysis of the greater trochanter in growing children. The use of an angled blade plate has been suggested to address the problem of the small femoral shaft in children (Table 3). This premise is supported by the findings in our current study of a good outcome of this procedure in the 3 patients who were treated with angled blade plates before the age of 12, although the blade plate had to be removed in one of the 3 patients due to complaints of the iliotibial tract. An allogeneic allograft may also be considered in pediatric patients with lesions of the femoral neck and no other risk factors. Regardless of the choice of treatment, it is important to appreciate that the risk of failure or of recurrence and the need for revision surgery is high in young and growing patients and that in pediatric patients there is a clearly unmet need for a tailored device providing stability and preventing further deformation of the femur.

## Discussion

In this study we evaluated the clinical outcome of two types of surgical interventions for fibrous dysplasia of the proximal femur using angled blade plates and intramedullary nails. Although in our cohort, surgery was undertaken at a median young age of 20 and one in two of the patients studied were aged 20 years or younger, data on skeletal maturity were not available, so that data from our cohort do not allow firm recommendations for choice of surgical intervention to be drawn for children with potential remaining substantial growth. Notwithstanding, our findings from this relatively young cohort of patients demonstrate that both modalities adequately maintained the immediate postsurgical improvement in FNSA, resulted in good clinical outcomes as regards function and pain and prevented fractures overall, although two patients did develop a pathological fracture distal to the implanted angled blade plate. These pathological fractures are likely to have been due to a stress riser effect in a fibrous dysplasia lesion, most probably as a result of the blade being not long enough to cover the whole area of the lesion. Angled blade plate implants were also associated with persistent complaints of the iliotibial tract in 7 patients with monostotic disease and relatively small fibrous dysplasia lesions, which necessitated removal of the angled blade plate with no further complications and good outcome at the end of follow-up.

The management of fibrous dysplasia has been very challenging ever since the disease was first described, not the least because of the wide and heterogeneous clinical spectrum of fibrous dysplasia, but also because of its variable association with extraskeletal manifestations [1–3, 10]. Nowhere is this more true than in the management of fibrous dysplasia of the proximal femur. A fibrous dysplasia lesion of the proximal femur is thus known to be associated with more pain, more fractures and especially with more deformity than any other skeletal localization of the disease [9, 10, 28, 38]. Surgical approaches to the treatment of a fibrous dysplasia lesion of the proximal femur must therefore not only include the treatment and prevention of pathological fractures, but also aim at preventing the progression of a varus deformity. The rare nature of fibrous dysplasia has resulted in the outcome of different treatment modalities having been so far evaluated in only small and often heterogeneous series of patient, in whom different surgical interventions were performed, usually at the treating physician’s discretion rather than being based on a high level evidence or on consensus guidelines. Of the several surgical interventions performed in the management of fibrous dysplasia over the past few decades, a number had to be abandoned because of failure of the procedure or high rates of complications. We reviewed here the outcomes of most of the surgical interventions reported in the literature, which have been used in the management of fibrous dysplasia of the proximal femur, including bone grafting, intramedullary nails, angled blade plates and dynamic or compression hip screws. We also reviewed published literature on the treatment of fibrous dysplasia of the proximal femur in pediatric patients.

### Proposed surgical treatment algorithm for the management of fibrous dysplasia of the proximal femur

**Fig. 4:**
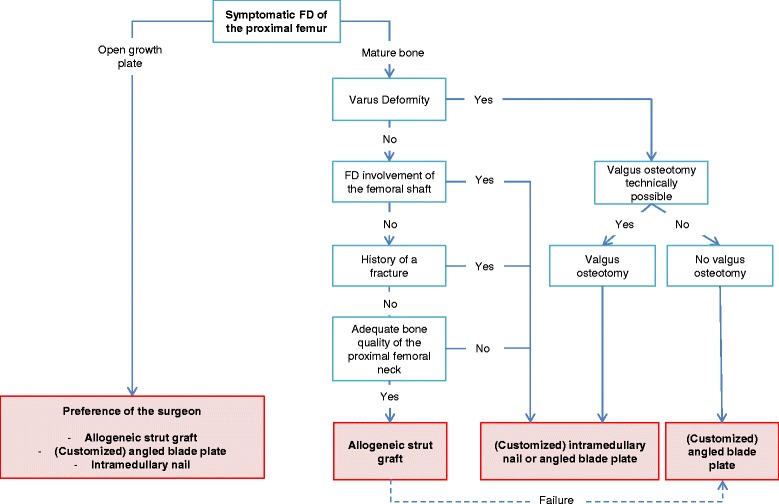
Proposed individualized, patient-tailored algorithm for the surgical management of fibrous dysplasia of the proximal femur

Based on a review of published literature and on findings from our combined cohort study, we propose the following algorithm for the surgical management of fibrous dysplasia of the proximal femur (Fig. 4). In this proposed treatment algorithm there is a place for allogeneic strut grafting, albeit limited to cases without a previous fracture, without a fibrous dysplasia lesion extending to the femoral shaft, without a deformity of the femur requiring a valgus osteotomy and with adequate bone-stock proximally in the femoral neck [17]. In case of failure of the allogeneic strut graft, revision surgery with an angled blade plate is preferred over the placement of an intramedullary nail, as the introduction of a blade is easier to perform in a femoral neck with remnants of strut grafts. Internal fixation with either an intramedullary nail or an angled blade plate is preferred in patients with risk factors associated with the placing of an allogeneic strut graft. Using either of these devices, it is imperative that the fibrous dysplasia lesion is completely bridged especially in the femoral head, with adequate, bipolar fixation of the implant ends in healthy bone with either the blade of the angled blade plate or the screw of the intramedullary nail. Angled blade plates should also be adequately submerged into the cortex to prevent the development of complaints of the iliotibial tract. In case of severe deformity of the femur without the possibility of a valgus osteotomy, a customized blade plate is the implant of choice. The use of dynamic or compression hip screws and TENs is not recommended in fibrous dysplasia of the proximal femur.

## Conclusion

Fibrous dysplasia of the proximal femur can be adequately and safely treated with angled blade plates, intramedullary nails or allogeneic strut grafts, provided that these are used according to the specific characteristics of the individual patient. Fibrous dysplasia of the proximal femur remains a challenging entity, but continuous improvements in a variety of treatment options have paved the way towards a more favorable clinical outcome. Based on published literature, decades of experience from 2 expert centers in Austria and the Netherlands and data from a combined cohort in this study we propose an individualized, patient-tailored algorithm for the surgical management of fibrous dysplasia of the proximal femur, taking into account different treatment modalities and associated factors that play a role in the outcome of the different implants. Future research should focus on the development of implants that meet the specific needs of the challenging pediatric and adult patients with fibrous dysplasia of the proximal femur.

## Additional file

Additional file 1: Revision surgery after allogeneic strut grafting in fibrous dysplasia of the proximal femur. (DOCX 18 kb)

## Abbreviations

cAMP: Cyclic adenosine monophosphate
CHS: Compression Hip Screw
DHS: Dynamic Hip Screw
FNSA: Femoral-Neck-Shaft-Angle
G_s_α: Alpha subunit of the G-protein
TEN: Titanium-Elastic-Nails
