# Supplementary material for: Individualized approach to the surgical management of fibrous dysplasia of the proximal femur
Source: Orphanet J Rare Dis. 2018 May 2;13:72. doi: 10.1186/s13023-018-0805-7 (PMC5932767; doi:10.1186/s13023-018-0805-7)
Supplement: Supplementary file 1 — Revision surgery after allogeneic strut grafting in fibrous dysplasia of the proximal femur. (DOCX 18 kb) [file 13023_2018_805_MOESM1_ESM.docx]

**Additional file 1**

Allogeneic cortical strut grafts represent an attractive option for the treatment of fibrous dysplasia lesions of the proximal femur, provided that patients dot not have any of the previously described risk factors for failure of the procedure.^1^ There are scarce data on possible treatment options after failure of allogeneic strut grafts in fibrous dysplasia of the proximal femur. We evaluated our cohort of 29 patients who were treated with these types of grafts for failure rates after consecutive different surgical interventions.

**Patients and Methods**In a retrospective study design, we included 29 patients (15 male) with allogeneic strut grafts without osteotomy and/or osteosynthesis between 1980-2013 and with a minimal follow-up of two years after surgery. Mean age a time of surgery was 22,9 years (5-50 years) and the primary outcome of this study was failure, measured as revision surgery for fracture, progressive deformity or progressive resorption of the graft.

**Results**After a median follow up after surgery of 9 years (2-37yrs) 14 patients (48%) needed a reoperation after the first ACGS. Patients presenting with a pathological fracture before index ACGS had an increased risk for failure (p<0.05). Mean age at time of failure was 27 years (14-42), median time to failure was 4.5 years (0-20 years) and gender was evenly distributed. Failure mechanisms were resorption (50%), fracture (43%) and one patient needed a reoperation as a result of progressive deformity of the proximal femur. On average patients with a reoperation had 1.9 reoperations (± 1.4SD) compared to 0.9 (± 1.4SD) reoperations in the whole group of FD patients. Seven patients were treated again with allogeneic strut grafts, two with intramedullary nails, four had severe bowing of the femur and received a blade plate (3 custom made) and one patient was treated with prevot pins. Four out of seven patients with consecutive allogeneic strut grafts and the patient with prevot pins needed at least one more revision surgery as a result of consecutive failure. All patients who received a blade plate (4) or intramedullary nail (3) had improvement of pain and mobility and did not need another reoperation.

**Conclusion**These data show that failure after allogeneic strut grafts in general occurs in the first three years after surgery. Failure of allogeneic strut grafts in fibrous dysplasia of the proximal femur appears to be a risk factor for failure of a consecutive intervention with the use of these grafts. We therefore recommend treating patients with primary failure of allogeneic strut grafts with an additional osteosynthesis of the proximal femur.

1. Majoor BC, Peeters-Boef MJ, van de Sande MA, Appelman-Dijkstra NM, Hamdy NA, Dijkstra PD. What Is the Role of Allogeneic Cortical Strut Grafts in the Treatment of Fibrous Dysplasia of the Proximal Femur? *Clinical orthopaedics and related research.* Mar 28 2016.
